# Kinetic mechanism of wheat straw pellets combustion process with a thermogravimetric analyser

**DOI:** 10.1016/j.heliyon.2023.e20602

**Published:** 2023-10-02

**Authors:** Bidhan Nath, Guangnan Chen, Les Bowtell, Elizabeth Graham

**Affiliations:** a School of Agriculture and Environmental Science, University of Southern Queensland, Toowoomba, QLD, 4350, Australia; b School of Engineering, University of Southern Queensland, Toowoomba, QLD, 4350, Australia; c Physical and Mechanical properties Laboratory, Central Analytical Research Facility, Queensland University of Technology, Brisbane, QLD, 4000, Australia

**Keywords:** Combustion, Wheat straw pellet, Thermogravimetric analyser, Derivative thermogravimetric analysis, Heating rate, Model-based methods

## Abstract

In this study, the combustion characteristics of two wheat straw pellets (WSP) (T_1_: 100% wheat straw and T_5_: 70% wheat straw; 10% sawdust, 10% biochar; 10% bentonite clay) were performed at a heating rate 20 °C/min under a temperature from 25 to 1200 °C in air atmosphere. A thermogravimetric analyser (TGA) was used to investigate the activation energy (*E*_*α*_), pre-exponential factor (*A*), and thermodynamic parameters. The DTG/TG profile of WSP was evaluated by model-free and model-based methods and found the model-based method was suitable for WSP thermal characterisation. The result demonstrates that the thermal decomposition occurred in four stages, comprising four consecutive reaction steps. A→B→C→D→E→F. Further, the model-based techniques were best fitted with kinetic reaction models like *Cn* (nth^-^order reaction with auto-catalyst), *F*n (reaction of nth order), *F2* (second-order phase interfacial reaction) and *D3* (diffusion control). The average *E*_*α*_ for *Fn, Cn, D3* and *F2* models were 164.723, 189.782, 273.88, and 45.0 kJ/mol, respectively, for the T_1_ pellets. Alternatively, for T_5_ pellets, the *A* was 1.17E+2, 1.76E+16, 5.5E+23, and 1.1E+3 (1/s) for *F2, D3, Cn* and *Fn* models. Overall, the thermodynamic properties showed that WSP thermokinetic reactions were complex and multi-point equilibrium, indicating a potentiality as a bioenergy feedstock.

## 1 Introduction

Globally, biomass is a promising source of bioenergy and has attracted increasing attention [[1], [2], [3]]. Agricultural waste is primary biomass with poor-quality solid fuel characteristics [4]. Lower density, a low calorific value, and higher ash content are the main drawbacks of agricultural waste/lignocellulosic straws [5]. In addition, straw is usually less expensive and widely available, mainly generated from field crops. Wheat straw is available globally among the different types of agricultural waste [6]. This research used wheat straw (WS) pellets that are blended with different additives. The additives can change the poor-quality wheat straw into a higher-valued solid fuel [[7], [8], [9]].

A large amount of scientific literature is available on wheat straw co-combustion with TGA (thermogravimetric analyser) (Table 1). However, most research has been conducted on coal blended with biomass [6,10,11]. Xinjie, Singh [12]. Hence, coal could increase wheat straw combustion efficiency [13], but coal production and use are an environmental concern. In this context, blending available additives aside from coal with WS could be an alternative for generating thermal energy while sustaining environmental sustainability.

**Table 1:** Thermokinetic analysis for biomass.

Materials	Technology	Findings	Reference
Sawdust pellet	TGA and Fluidised-bed reactor for combustion	Combustion indices, ignition, and burnout temperature CO, CxHy and NO emission	[14]
Biomass	Machine learning model	Chemical, thermochemical and biochemical conversion processes and life cycle assessment	[15]
Biomass	Machine learning and artificial neural network (ANN)	Combustion index	[16]
Caol and duckweed biomass	Combustion (TGA	Ignition and burnout time	[17]
Biomass, coal and blends	Artificial neural networks (ANN)	Activation energy	[18]
Lignocellulosic biomass	TGA and FTIA analysis	Kinetic reaction and char structural transformation	[19]
Sawdust	Combustion (TGA)	Thermal stability and activation energy	[20]
Biomass-plastic blends	Oxy-fuel combustion (TGA)	Synergistic behaviour Comprehensive combustion index	[21]
Torrefied pine wood	Combustion (TGA)	Thermal characteristics	[22]
Napier grass	Combustion and Pyrolysis (TGA)	Thermal characteristics	[23]
Wood biomass pellet	Combustion (TGA)	Combustion characteristics	[24]
Palm kernel shell, African bush mango wood, and shell	Combustion (TGA)	Oxidation characteristics	[25]
Corn stover	Combustion and Pyrolysis (TGA)	TGA characteristics	[26]
Rice straw and pine sawdust	Combustion (TGA)	Pyrolysis kinetics characteristics	[27]
Wheat straw and plastic	Combustion and Pyrolysis (TGA)	Pyrolysis yield	[28]
Mustard	Combustion (TGA)	Biochar, bio-oil, and hydrocarbon gases	[29]
Corn straw powder, poplar wood chip, and rice husk	Combustion and Pyrolysis (TGA)	Pyrolysis and combustion characteristics	[30]
Barley straw, miscanthus, waste wood, wheat straw, willow, and wood pellet	Combustion (TGA)	Thermal and kinetic analysis	[11]
Biomass pellet (pine wood and corn straw)	Combustion (TGA)	Combustion kinetics and mechanism	[31]

The thermal performance and co-combustion kinetics between wheat straw and sewage sludge and their blends were also studied empirically using TGA, and the results showed a substantial synergetic interaction in high-temperature zones [32]. Paniagua, García-Pérez [33] conducted a thermal study of wheat straw and poplar wood blends and found the combination achieves the best combustion characteristics indexes. El-Sayed and Khairy [34] experimented with the burning and emissions of pelletised wheat straw in high-temperature airflows. This study only investigated the decomposition behaviour, but the thermokinetic parameter did not identify. Ríos-Badrán, Luzardo-Ocampo [35] recently investigated manufacturing and characterising pellets from wheat straw and rice husk through TGA and lab experiments. They contend that biomass mixtures enhance the pellet's quality and combustion properties. Barley straw, waste wood, wheat straw, willow, miscanthus, and wood pellets were studied using thermal and kinetic analyses by Sher, Iqbal [11]. They discovered an inverse relationship between activation energy and reactivity. Overall, the literature review suggests that thermokinetic combustion studies of additive blends of wheat straw pellets and individual wheat straw pellets are still rare [14]. Therefore, research on the combustion of WSP is essential to address combustion properties for reactor design and biomass-to-energy transformation.

Biomass thermochemical conversion involves intricate physiochemical mechanisms [36]. Understanding solid-state degradation kinetics and heterogeneous reaction processes is essential for TGA [37]. TGA is a powerful, commonly used tool for measuring the thermogravimetric (TG) profile based on mass changes function of time or temperature [38,39]. Inversely, the derivative thermogravimetry curve (DTG) is also useful to observe the act of temperature and time for reaction rate. The non-isothermal and isothermal models are generally used for the thermal analysis of the TG/DTG profile. The non-isothermal technique has recently been preferred because it has less experimental noise and is less complicated than the isothermal method [40]. Recently, Wei, Luo [41] and Ni, Bi [42] have invested in the thermogravimetric of co-combustion coal, sewage sludge and biomass using machine learning. However, no study was found on wheat straw pellet combustion based on machine learning as well as TGA analysis. Therefore, WSP's thermokinetic properties optimisation study is very essential [43].

In addition, the isoconversional (differential or integral) methods can accurately determine the apparent activation energy without knowing the reaction model beforehand. Model-free and model-fitting approaches are the main ways of estimating non-isothermal solid-state kinetic data [44]. The model-free approach is suitable for a one-step reaction. Alternatively, model-fitting is appropriate for complex biomass decomposition with one or multi-point reactions [45]. Therefore, this study used model-free and model-based methods to investigate and contrast the thermal decomposition (TG/DTG curves) pattern and determine the most suitable model. The different kinetic approaches, including model-based (of nth order reaction with autocatalysis: *C*_*n*_, and the reaction of nth order (*F*_*n*_)) and model-free (Friedman, OFW, and KAS) methods, were also utilised to characterise the kinetic parameters. Table 2 summarises a few kinetic reaction models often used in solid-state reaction kinetics.

**Table 2:** Common reaction models [[46], [47], [48], [49]].

Reaction	Model name	Code	Functions
Chemical reaction	Zero-dimensional phase boundary	R_0_	*0*
	First-dimensional phase boundary	R_1_	*f = e*
	Two-dimensional phase boundary	R_2_	*f = 2e*^*1/2*^
	Three-dimensional phase boundary	R_3_	*f = 3e*^*2/3*^
Phase interfacial reaction	First-order reaction	F_1_	*f = e*
	Contracting cylinder (Second-order)	F_2_	*f = e*^*2*^
	Contracting sphere (Third-order	F_3_	*f = e*^*3*^
	Random nucleation (Fourth-order)	F_4_	*f = e*^*4*^
	Reaction of *n*th order	F_n_	*f = e*^*n*^
Diffusion models	One-dimensional diffusion	D_1_	*f = e*^*0.5/p*^
	Two-dimensional diffusion (Valensi model)	D_2_	*f = -1/*ln*(e)*
	Diffusion control (Jander model)	D_3_	*f = 1.5e*^*2/3*^*/(1-e*^*1/3*^*)*
	Diffusion control (Ginstling model)	D_4_	*f = 1.5/(e*^*−*^*^1^*^*/3*^*-1)*
Nucleation and growth models	Two-dimensional nucleation, according to Avarami -Erofeev	A_2_	*f = 2e.[-ln(e)]*^*1/2*^
	3D nucleation, according to Avarami- Erofeev	A_3_	*f = 3e.[-ln(e)]*^*2/3*^
	*n*-Dimensional nucleation according to Avrami–Erofeev	A_n_	*f = n.e.[-ln(e)]*^*(n−*^*^1^*^*)/n*^
Auto-cat reaction	Reaction of 1st order with autocatalysis by product	C_1_	*f = e. (1* + *AutocatOrder.P)*
	Reaction of nth order with autocatalysis by product	C_n_	*f = e*^*n*^*. (1* + *AutocatOrder.P)*
Nomenclature			
BC	Bentonite Clay	LHV	Lower Heating Value
BD	Bulk Density	M	Mass
BioC	Biochar	SD	Sawdust
CFD	Computational Fluid Dynamics	TA	Thermal Analysis
db	Dry Basis	MC	Moisture content
DTG	Derivative Thermogravimetric	TGA	Thermogravimetric Analyser
FC	Fixed Carbon	TG	Thermogravimetric
HHV	Higher Heating Value	VM	Volatile Matters
A	Pre-exponential factor	wb	Wet Basis
α	Degree of conversion	WL	Weight Loss
$E_{\alpha }$	Activation of energy	WS	Wheat Straw
Δ G	Gibbs free energy	WSP	Wheat Straw Pellet
Δ H	Enthalpy/latent heat enthalpy	T_i_	Ignition temperature
Δ S	Entropy	T_b_	Burnout temperature
β	Heating rate	R	Universal gas constant
$K_{\beta }$	Stefan-Boltzmann constant	K	Reaction rate constant
n	Reaction order	h	Planck constant

The present work investigates the wheat straw pellets (with (T_5_) and without (T_1_) additives) combustion for kinetic behaviour using TGA. The main intentions of the present work were: (a) to use model-free and model-fitting methods to examine and contrast the thermal decomposition techniques; (b) determination of burning profile parameters (*E*_*α*_*, A*, and *f(α)*)*;* and c) determine of thermodynamic parameters. These kinetic parameters could use as input data in Computational Fluid Dynamics (CFD) modeling for designing a reactor and analysing the pellets' energy conversion.

## 2 Materials and methods

### 2.1 Test sample and sample preparation

The pellet was a cylindrical solid fuel made from wheat straw with (T_5_) and without (T_1_) additives. The materials' chemical analysis results are shown in Table 3, which was done in the previous study by Nath, Chen [9].

**Table 3:** Test sample physicochemical characteristics.

Sample /Pellets	Proximate analysis, %, as received, dry basis				Ultimate analysis, %, dry basis					Physical parameter		
	MC	VM	FC	Ash	C	H	N	S	O*	Average length, mm	Mean diameter, mm	Bulk density, kg/m^3^
T_1_	6.20	75.61	11.10	7.09	44.32	4.90	0.56	0.11	50.11	22.0	8.21	244.79
T_5_	3.50	53.03	31.60	11.87	45.87	6.30	0.72	0.21	46.90	37.0	8.13	607.40

Note: MC = Moisture content; VM = Volatile matters; FC = Fixed carbon; * by difference.

T_1_: 100% wheat straw.

T_5_: 70% wheat straw; 10% sawdust, 10% biochar; 10% bentonite clay.

The TGA samples need to be ground to increase the surface area [50] and conversion efficiency [51]. The samples were dried at 105 °C for 24 h in an oven and then ground into a powder with an average 1 mm particle size. Then, the crushed sample was sieved to ensure a uniform size of particles. A dummy test was done on each heating rate to avoid systematic error and baseline information.

The same sample size (weight) was used for each treatment to ensure accurate experimental results [52]. Regarding this issue, we considered a 50 mg sample as an initial weight for each run. However, the sample holding capacity of the TGA pan was 8.75–9.75 mg. The sample was burned in an Alumina-based pan and used a lid to be covered to create the best possible heat transmission conditions. The experiment was repeated three times to maintain the precision and reproducibility of the analysis. The collected data's mean value was used for the current study.

### 2.2 Thermogravimetric analyser

TGA is the most common technique used to investigate fuels' thermal behaviour and kinetics of carbonaceous materials as a function of temperature (non-isothermal) and time (isothermal) [[53], [54], [55]].

The STA 449F3 Jupiter TGA (Erich NETZSCH GmbH & Co. Holding KG, Germany) was used to measure and record the dynamics of the constant mass loss of the samples with increasing temperature and time [56]. This Jupiter TGA equipment comprises a furnace, sample pan, precision balance, gaseous supply system, and data collection system [57]. During the experiment, the feedstock was combusted in the control zone under a pressure of 0.1 MP. Air was used as the carrier gas and kept at a 50 ml/min steady flow rate.

In the present study, the kinetic triplets were derived using state-of-the-art kinetic software NETZSCH Proteus 8.0 (NETZSCH-Gerätebau GmbH) for WSP pyrolysis under dynamic conditions. NETZSCH software allows analysing of temperature-dependent chemical processes [49]. A computer automatically controls the entire process, records the mass changes, and draws a weight loss curve, resulting in a kinetic model or method describing the experimental data under applied temperature conditions.

### 2.3 Data analysis and kinetic parameters

The mass loss (TG curves) was employed to estimate the degradation of pseudo-component (lignin, hemicellulose, and lignin) [58]. In contrast, the degradation rate was assessed by DTG profiles [59]. The TGA data were obtained from the STA 449F3 Jupiter TGA built-in computer at 20 °C/min heating rates, and temperatures ranged from 25 to 1200 °C.

Thermo-kinetic properties of feedstock and operating conditions can significantly influence the efficiency of a reactor and conversion performance [60]. According to the Arrhenius law, three variables (*E*_α_, *A*, and (***α***)), are highly relevant in the study of thermo-kinetic decomposition. Generally, these three parameters are referred to as “kinetic triple” and represent the thermal breakdown [61].

### 2.4 Kinetic theory

A kinetic modelling study using thermogravimetric analysis data can explore the feedstock decomposition behaviour and biomass processing mechanism [62]. The conversion or thermal decomposition rate is a heterogeneous reaction and can be expressed as a single-step kinetic equation [61,63]. This formula (Eq. 1) represents the mass conversion rate of two functions (k(T)andf(α)) and can be denoted as [64]:

(1) $$\frac{d\alpha }{dt}=k\left(T\right)f\left(\alpha \right)$$

Where f(α) is conversion model depends on the actual reaction mechanism [65], *α* is the degree of conversion and k(T) the reaction rate at absolute temperature T.

The fraction (α) data is commonly used for the kinetic model of that material decomposition. The conversion degree (α) was defined as (Eq. (2)):

(2) $$\alpha =\frac{w_{0}-w}{w_{0}-w_{f}}$$

Where α=reactantdecompositionfractionatthetime (t),

${w,w}_{0}{,w}_{e,}w_{f}$ = the sample's initial, actual, and final weights (gm), respectively, and

*n* = the reaction order.

Commonly, the Arrhenius law is used to calculate biomass combustion and pyrolysis kinetic parameters. This law is also important in obtaining information about the reaction rate. The Arrhenius law can be expressed mathematically (Eq. (3)) [66,67]:

(3) $$k\left(T\right)=A\;e^{\left(-\frac{E_{\alpha }}{RT}\right)}$$

**Table:** 

Where	
*k* = reaction rate constant, 1/min	$E_{\alpha }$ = activation of energy, kJ/mol
A = pre-exponential factor, 1/min	T = Absolute temperature, K
R = universal gas constant (8.314), kJ/K.mol	

The following equation (Eq. 4) can be expressed using Eqs. (1), (3) for the non-isothermal reaction process with a linear/constant heating rate [68]:

(4) $$\frac{d\alpha }{dT}=\frac{A}{\beta }\;e^{\left(-\frac{E_{\alpha }}{RT}\right)}f\left(\alpha \right)$$

Where the heating rate, $\beta =\frac{dT}{dt}$.

Various models have been used to explore the kinetic parameters through a single-step equation [69,70] two main categories of empirical models (model-fitting and model-free methods) are commonly used [71].

### 2.5 Model-free analysis

The kinetic analysis based on an iso-conversional technique is usually referred to as “model-free [72]. The model-free techniques are straightforward and can identify multi-step processes more accurately. This method gives information only on reactant (*A*) and product (*B*) and no other information on intermediate steps or products [73]. Hence this analytical method can mostly be done on paper or an Excel sheet. However, it only works for mixtures or competitive or highly overlapping steps [74]. The process describes only one chemical, Eq. (5) (Arrhenius equation) [75].

(5) $$\frac{d\alpha }{dT}=A\left(\alpha \right)\;.f\left(\alpha \right).\;\exp\;\left(-\frac{E_{\alpha }}{RT}\right)$$

Here (α) and A(α) are unknown, while A(α) can be found only with the assumption of f(α).

It is not so easy to describe this equation with one value. Therefore, this method computes $E_{\alpha }$ from a series of TG data sets via constant heating rate figures or/and temperatures [61,71,76]. In addition, the method is based on some assumptions and kinetic reactions and does not depend on specific models [61]. The data must satisfy specific assumptions to validate any model by model-free techniques, such as [49]:

- Only one kinetic equation, for example, Reactants A → products B.
- $E_{\alpha }$, and A depends on α (degree of conversion).
- The reaction rate at the same conversion is only a function of temperature.
- The total effect (total mass loss or total peak area) must be the same for all curves.
- Changes in mechanism should be at the same conversion value.

There are several model-free methods, including the (i) Kissinger approach [77], (ii) Kissinger-Akihara-Sunose (KAS) [78], (iii) Friedman techniques [79], (iv) Flynn-Wall [80] and (v) Flynn-Wall-Ozawa (FWO) [81]. Vyazovkin, Burnham [47] noted that the most accurate techniques are KAS and FWO for kinetic parameter evaluation. However, the Friedman approach uses a differential tool [79]. At the same time, the KAS and FWO are the two frequently applied integral techniques [82]. They are widely used for many purposes [83,84], but all are based on some presumptions.

### 2.6 Model-based analysis methods

The model-based kinetic method analyses complex chemical processes with multiple reaction steps (https://kinetics.netzsch.com/en). Each reaction step has its kinetic equation and a kinetic triple [85]. In addition, the model-based approach can display each reactant's reaction rate and concentration for each step [86]. Also, Karaeva, Timofeeva [87] mentioned that 95% of chemical reactions are multi-stage during thermal conversion. Hence, model-based kinetics is effective for comprehensively analysing chemical reactions. However, before examining the thermal data, the pre-requisite assumption of the model-fitting method needs to be acknowledged. The assumptions for model-based kinetic analysis are as follows [71].

- The reaction comprises several basic reaction steps with their kinetic reaction equations.
- All kinetic parameters are constant values.
- The total signal is the sum of the signals of the single reaction steps having their weight.

Kinetics Netzsch Proteus software can develops the best kinetic model that precisely captures the heterogeneous process using reliable, cutting-edge mathematical computations to determine kinetic triple. Two- and three-stage reaction models describe multi-stage reaction systems [88]. Following “International Confederation for Thermal Analysis and Calorimetry (ICTAC)” recommendations, one should construct a kinetic model that includes the least number of stages and corresponds to a significant experimental range [87]. The following Eq. (6) can define the general reaction rate for individual reaction steps [75].

(6) $$\text{Reaction}\;\text{rate}\;\left(j\right)=\frac{d\left(a\;\to \;b\right)}{dt}=A_{j}*f_{j}\left(e_{j}p_{j}\right)*\exp\;\left(-\frac{E_{\text{Aj}}}{RT}\right)$$

Where $f_{j}\left(e_{j}p_{j}\right)$ = function of reaction type, $e_{j}$ = initial reactant concentration,

$p_{j}$ = product concentration, $A_{j}$ = pre-exponential factor, 1/s
$E_{A}$ = activation energy, and *j* = number of specific reaction steps.

The literature suggests that kinetic triple estimation depends on the user, as different findings might be found for the same data set [89]. It relies on the selection of the model and the reaction types (i.e., independent, consecutive, or competitive) [90]. This thermal analysis used the Kinetics NETZSCH with Proteus 8.0 software multi-step analytical engines, which is an excellent tool for model-based and model-free analysis. This study used the following theoretical model to perform the multi-step kinetic model (Eqs. (7) and (8)) [[91], [92], [93]].

(7) $$n^{\text{th}}\;\text{order}\;\text{reaction}\;\text{with}\;\text{autocatalysis}\;\left(C_{n}\right),\frac{d\alpha }{dt}={Ae}^{\frac{E_{\alpha }}{RT}}\;{\left(1-\alpha \right)}^{n}\;\left(1+k_{cat}\;\alpha \right)$$

(8) $$n^{\text{th}}\;\text{order}\;\text{reaction}\;\left(F_{n}\right)=\frac{d\alpha }{dt}={Ae}^{\frac{E_{\alpha }}{RT}}\;{\left(1-\alpha \right)}^{n}$$

where n = Reaction order, *k*_*cat*_ = Catalytic rate constant.

### 2.7 Thermodynamic analysis

Biomass combustion requires information on thermodynamic parameters [94]. The essential thermodynamic parameters (Enthalpy: ΔH, entropy: ΔS, and Gibbs free energy: ΔG) are often used to characterise thermal behaviour [48,95]. The thermodynamic parameters depend on the combustion process efficiency and the measurement of heat [96]. Moreover, energy calculation and the process feasibility determination depend on the thermodynamic analysis results. To determine Δ H, Δ G, and ΔS from kinetic parameters the following measures can be used Eqs. (10), (11) [97,98]:

(9) $$\Delta H=E_{a}-RT$$

(10) $$\Delta G=E_{a}+RT_{m}\;\ln\left(\frac{K_{\beta }T}{hA}\right)$$

(11) $$\Delta S=\frac{\Delta H-\Delta G}{T_{m}}$$

Where $K_{\beta }$ = Boltzman constant (1.38 *10^−23^), m^2^.kg/s.k

*h* = Planck constant (6.626*10^−34^), m^2^.kg/s.

$T_{m}$ = Maximum temperature at which maximum decomposition occurred, K

*R* = Universal gas constant (8.3145), J/mol.K.

For the calculation of ΔH, ΔS, and ΔG, the pre-exponential factor and activation energy data were taken from NETZSCH Kinetics software at a particular conversion for 20 °C/min. The thermodynamic parameters were calculated based on the highest temperature where the maximum decomposition occurred and acquired from the DTG profile [99,100].

## 3 Results and discussion

### 3.1 Kinetic study of wheat straw pellet

**Fig. 1:**
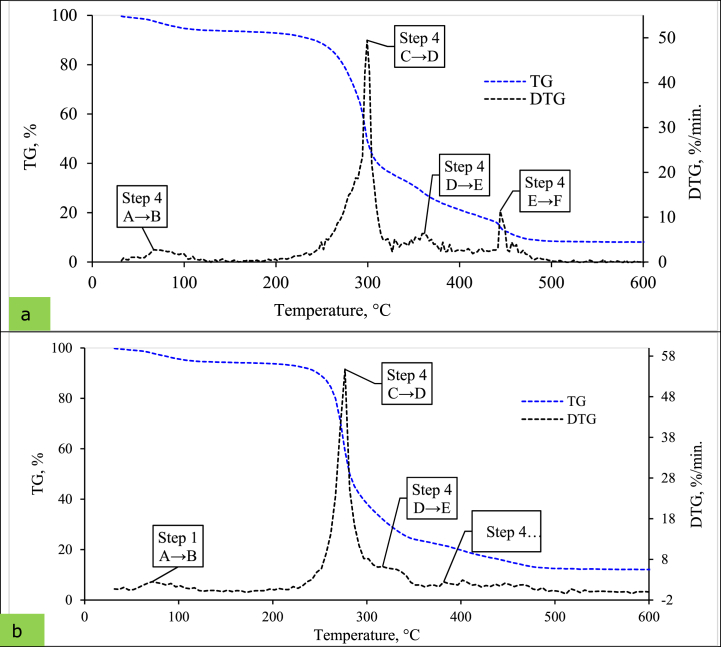
Thermogravimetric (TG) and Derivative thermal analysis (DTG) profile for (a) T_1_ and (b) T_5_ pellet at 20 °C/min heating rate.

The TGA is a technique that measures the different reaction steps concerning temperature and conversion rate. This advanced technique investigates reaction rates because of simulated curves representing reaction steps (Fig. 1a,b). As the figure shows, the DTG curve suggests four different reaction steps for four identifiable peaks during the combustion process [101]. Alvarez, Pizarro [102] conducted a study to determine the kinetic parameters for 28 different biomass and found that most samples followed the four peak degradation patterns (DTG curve). These findings are consistent with the results obtained in the present study.

During combustion, both pellets followed the same trend, and temperature differences were similar, but the reaction rates step to step. Step 1, denoted by A → B, is called the dehydration and desorption stage [103]. In this stage, moisture was evaporated below 110 °C while degraded hemicellulose. Alternatively, step 2 (C → D) is termed by the oxidation phase. In this stage, the hemicellulose and cellulose burned at a temperature range of 110∼350 °C, removing the volatile matter [49]. On the other hand, step 3 (D →E) is the main combustion phase, where the temperature varies from 350 to 650 °C and decomposed hemicellulose and cellulose of wheat straw pellets [71]. Step 4 (E →F) of the combustion process is named char combustion, where lignin decomposes and remains the carbon-enriched ash/charcoal. In these steps, the temperatures reached over 650 °C. These findings agree with the previous researchers, even though they considered different types of biomass [90,102].

The thermal degradation behaviour (TG curve) of pellets T_1_ and T_5_ is also presented in Fig. 1 using a heating rate of 20 °C/min. It was observed that both pellets exhibited a similar trend in terms of mass loss. As is commonly known, the thermal degradation of most biomass materials typically occurs in three stages [104]. Similarly, the current study followed this pattern, with a gradual loss of mass observed between ambient temperature and 250 °C, a sudden mass loss in the temperature range of 250–475 °C and a steady mass loss between 475 and 600 °C. These findings align with the results of previous research studies [105], indicating consistency and agreement in the observed thermal degradation of biomass samples.

### 3.2 Evaluation of wheat straw pellet profile through kinetic model

**Fig. 2:**
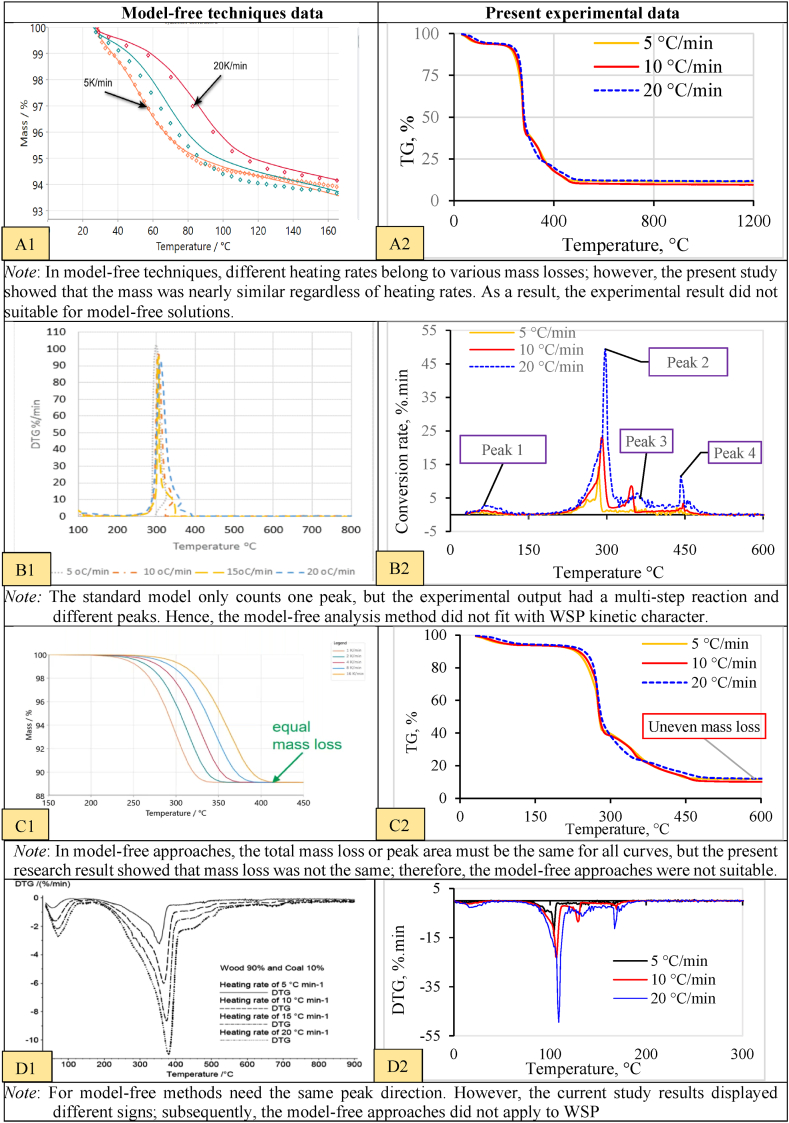
(A1 -D2). Evaluation of wheat straw pellet combustion characteristics Note: A1 and C1 = TG curve (model-free), A2 and C2 = TG curve (model-based) C1 A11 B1 and D1 = DTG curve (model-free), B2 and D2 = DTG curve (model-based). *Curve visualisation from an inbuilt computer with TGA.

Thermal decomposition data are frequently analyzed using various kinetic models, including model-free and model-based methods [106]. Typically, TG and DTG profiles were used to synthesise the thermo-kinetic properties. Therefore, the primary criteria of the model were used to assess the present WSP combustion results. Assessment of WSP combustion properties with the standard model data is shown in Fig. 2 (A1, A2, B1, B2, C1, C2, D1, D2).

Any biomass that has followed the single-step reaction in conversion that applies to model-free and model-based techniques. The model-free approach is best for a single reaction [71]; however, the model-based approach applies to both single and multi-phase reactions [89]. From the review literature, the lignocellulosic biomass transformation process (kinetic mechanism changes) follows complex reactions [45]. In addition, the conversion of lignocellulosic biomass like wheat straw has filled the multi-point direction [107], where model-free analysis provides ambiguous and misleading results. However, some researchers consider single-step reactions for studying the lignocellulosic biomass [62,94,108].

The kinetic results interpretation for multi-point reactions using various methods varied greatly [6]. From the observation, the assumptions of the model-free technique did not fit the wheat straw pellets' thermal degradation profiles (TG/DTG curves). Instead, the model-free methods could produce contradictory kinetic results/misleading values of *E*_*α*_ and *A* due to the continuous changes during reactions. Therefore, this research considered only model-based approaches.

### 3.3 Combustion process analysis by model-based method

The DTG curves from Fig. 1 (a, b) show that the wheat straw pellets (T_1_ and T_5_) follow the multi-step reaction. Therefore, this study considers Kinetics *NETZSCH Proteus 8.0* Software's multi-step analytical engines. The Kinetics *NETZSCH* program allows model-free and model-based kinetic analyses on thermal measurements [86].

With this analysis and simulation, the Kinetics *NETZSCH* software estimated *E*_*α*_ and *A* and determined the compatible reaction mechanism [49]. After the simulation, used deep explanations to obtain combustion parameters (Table 4). The following differential equations could solve a single-phase reaction to a series of subsequent multi-phase combustion reactions, where the balance equation is as follows (Eq. (12)):

(12) $$\text{Mass}=\text{Initial}\;\text{mass}\;–\;\text{TotalMassChange}\times \left\{\text{Contribution}\;\left(a\to b\right)\times \int \left[\frac{d\left(a\to b\right)}{dt}\right]dt+\text{contribution}\;\left(c\to d\right)\times \int \left[\frac{d\left(c\to d\right)}{dt}\right]dt+\text{contribution}\;\left(d\to e\right)\times \int \left[\frac{d\left(d\to e\right)}{dt}\right]dt\right\}+\text{contribution}\;\left(e\to f\right)\times \int \left[\frac{d\left(e\to f\right)}{dt}\right]dt$$

**Table 4:** Reaction steps and Equations (13–18) during combustion of wheat straw pellets.

Model scheme	Reaction steps	Concentration equations
A-B C-D-E-F	A → B (step 1)	$\frac{da}{dt}=-\frac{d\left(a\to b\right)}{dt}$ = −$A_{1}.\;f_{1}\left(a.b\right).\;\exp\left(-\frac{E_{a1}}{RT}\right)$ … …. … …. … ….(13) $\frac{db}{dt}=\frac{d\left(a\to b\right)}{dt}$ = $A_{1}.\;f_{1}\left(a.b\right).\;\exp\left(-\frac{E_{a1}}{RT}\right)$ …. … … … … … … ….(14)
	C → D (step 2)	$\frac{dc}{dt}=-\frac{d\left(c\to d\right)}{dt}$ = −$A_{2}.\;f_{2}\left(c.d\right).\;\exp\left(-\frac{E_{a2}}{RT}\right)$ …… … … …. … …(15) $\frac{dd}{dt}=\frac{d\left(c\to \;d\right)}{dt}$−$\frac{d\left(d\to \;e\right)}{dt}$ = $A_{2}.\;f_{2}\left(c.d\right).\;\exp\left(-\frac{E_{a2}}{RT}\right)-A_{3}.\;f_{3}\left(d.e\right).\;\exp\left(-\frac{E_{a3}}{RT}\right)$ …...(16)
	D →E (step 3)	$\frac{de}{dt}=\frac{d\left(d\;\to \;e\right)}{dt}-$$\frac{d\left(e\to \;f\right)}{dt}$ = $A_{3}.\;f_{3}\left(d.e\right).\;\exp\left(-\frac{E_{a3}}{RT}\right)$−$A_{4}.\;f_{4}\left(e.f\right).\;\exp\left(-\frac{E_{a4}}{RT}\right)$ …… … (17)
	E →F (step 4)	$\frac{df}{dt}=\frac{d\left(e\to f\right)}{dt}$ = $A_{4}.\;f_{4}\left(e.f\right).\;\exp\left(-\frac{E_{a4}}{RT}\right)$ …… … … … … … …. (18)

**Note:***f*_*1*_*(a,b)* = $n_{1}$*a*${\left[-\ln\;\left(a\right)\right]}^{\frac{\left(n_{1}-1\right)}{n_{1}}}$; $f_{2}\left(c.d\right)=n_{2}$*c*${\left[-\ln\;\left(c\right)\right]}^{\frac{\left(n_{2}-1\right)}{n_{2}}}$.

#### 3.3.1 Analysis of the reaction model

The TGA/DSC curves of wheat straw pellet samples for the combustion experiment were divided into four main predominant stages (Table 4). Both pellets (T_1_ and T_5_) showed two distinct reactions: A → B and C →D →E→F (Table 4). The first reaction steps as A→B, where *A* was the reactant (sample as presented), and *B* was the product (dehydrated sample). Instead, the second reaction was as C→D→E→F, where *C* was the starting material, *F* was the final product, and *D* and *E* were the intermediate product (Table 5). The results obtained (four stages reaction mechanism) in the present study align with the findings reported by Mandal, Mohalik [90]. They carried out simultaneous thermal analysis (STA) of Indian coal, which involves the simultaneous measurement of both TGA and DSC (differential scanning calorimetry) curves to determine the reaction stages.

$$f_{3}\left(d.e\right)=n_{3}\;d\;{\left[-\ln\;\left(d\right)\right]}^{\frac{\left(n_{3}-1\right)}{n_{3}}}\;and\;f_{4}\left(e.f\right)=n_{4}\;d\;{\left[-\ln\;\left(d\right)\right]}^{\frac{\left(n_{4}-1\right)}{n_{4}}}$$

**Table 5:** Thermal reactions and kinetic parameter for wheat straw pellet.

Reaction step	Reaction type:	Equation	Sample	Activation energy (*E*_*α*_), kJ/mol	A, (1/s)	Reaction order	Contribution/slope
*1 (A*→*B)*	F2: Second order	$\frac{d\left(a\to b\right)}{dt}=A{*a}^{2}{*e}^{-\frac{E_{\alpha }}{RT}}$	T_1_	45.0	1.18E+2	–	0.080
			T_5_	45.034	1.17E+2	–	0.078
*2 (C*→D*)*	D3:3-D diffusion	$\frac{d\left(c\to d\right)}{dt}=A*1.5*\left[\frac{c^{\left(\frac{2}{3}\right)}}{1-c^{\left(\frac{1}{3}\right)}}\right]*e^{-\frac{E_{\alpha }}{RT}}$	T_1_	273.488	8.01E+9	–	0.573
			T_5_	418.935	1.76E+16	–	0.584
*3 (D*→E)	Cn: nth order	$\frac{d\left(d\to e\right)}{dt}=A*d^{n}*\left(1+A_{autocat}*e\right)*e^{-\frac{E_{\alpha }}{RT}}$	T_1_	189.782	1.54E+9	3.507	0.243
			T_5_	632.065	5.5E+23	9.288	0.197
*4 (E*→F)	Fn: nth order	$\frac{d\left(e\to f\right)}{dt}=A*e^{n}*e^{-\frac{E_{\alpha }}{RT}}$	T_1_	164.723	2.41E+4	1.194	0.104
			T_5_	115.470	1.1E+3	2.059	0.141

Note: $A_{autocat}$:0.010.

The 1st stage (A→B) was the pore gases releasing phase after the removal of internal moisture from WSP pores, and 2nd stage (*C* →*D* →*E*→*F*) was the sorption of oxygen within the WSP matrix by increasing mass gain. So, for kinetic modeling, it was simplified by assuming four different reaction steps, i.e., A→B corresponds to the dehydration and desorption stage, C→D represents the oxidation stage, D→E denotes the combustion stage, and E→F associates the burnout stage. Based on various peaks of the DTG curve developed, this kinetic approach (Fig. 1 (a, b)). This phase category was in the entire agreement presented results by Manić, Janković [49].

Overall, the combustion process of wheat straw pellet followed the four-step consecutive reaction process as A → B →C → D→E→F, where *A, B, C, D*, *E* and *F* represent the decomposition states. The reaction rate for decomposition for each step was given by $\frac{da}{dt}$. In the presented equations, total conversion, *α* = 1 = a + b + c + d + e + f, while the *a, b, c, d, e* and *f* represent *A, B, C, D, E* and *F* concentrations in the chemical model kinetics. Moreover, the consecutive mechanism follows: *a* = *(1- α*_*1*_*), b* = *(1- α*_*2*_*), c* = *(1- α*_*3*_*), d* = *(1- α*_*4*_*),* and *A*_*1*_*, A*_*2*_*, A*_*3*_*, A*_*4,*_ $E_{a1}$*,* $E_{a2},E_{a3}$ and $E_{a4}$ signifies pre-exponential factors and activation energy quantities linked to the first, second, third and fourth reactions steps. Moreover, *n*_1_, *n*_2_, *n*_3,_ and *n*_4_ are reaction orders associated with the autocatalyst's first, second, third, and fourth reaction steps.

#### 3.3.2 Analysis of Kinetic triple

The kinetic triplets are important for optimising industrial reactors and predicting reactions [105,109]. To find kinetic triplicates apply the model-based approaches assuming the nth order of reaction (*f(α)* = $e^{n}$). The four different consecutive reaction steps (A→B, C→D, D→E, E→F) as shown in Table 4 [75,110]. This thermal process combines or overlaps several mechanisms, including nucleation, diffusion, and interface. However, the reaction depends on the sample origin, processing, and experimental conditions [89]. The activation energy and pre-exponential factor derived by NETZSCH Proteus 8.0 software for each type of reaction step regarding T_1_ and T_5_ pellets are shown in Table 5.

In reaction step 1, hemicellulose content boosted the sequential mechanism, where *E*_*α*_ was 45.0 kJ/mol (lowest) for the T_1_ pellet. The highest *E*_*α*_ (273.488 kJ/mol) was observed in step 2, where the cellulose content enhanced the successive mechanism (Table 5). The lower Eα value typically indicated less energy was required to start the reaction, while the higher value meant that the reaction began gradually [111]. Alternatively, the *E*_*α*_ varies from 45.0 to 273.488 kJ/mol for T_1_ pellets. The *E*_*α*_ and *A* values fluctuate with the reaction stages, ensuring that a complex combustion reaction occurred during the entire combustion range that successfully decomposed the hemicellulose and cellulose. Lin, Cho [112] reported that the *E*_*α*_ varies from 48 to 282 kJ/mol for the rea°tion mechanism for the primary two-step. The present study results were slightly lower than the experimental reports of Lin, Cho [112], which might be the different feedstock, temperature difference, and heating rates.

The cellulose mostly decomposed in the second (step 2); however, in the third region (step 3), cellulose and lignin contributions were lesser [113]. The *E*_*α*_ and *A* values in step 3 were 272.488 kJ/mol and 22.804 (1/s), which noted the second-highest value for the T_1_ pellet. In step 4, the activation energy and pre-exponential factors were 164.723 kJ/mol and 10.089 (1/s), resulting in lignin decomposition with the probability of several dissipations of volatile reaction pathways. After step 4, found residual ash and tars as remaining materials.

Table 5 shows the *E*_*α*_ and *A* for each reaction step during the combustion of T_5_ pellets obtained from the NETZSCH Proteus 8.0 software. The lowest activation energy and pre-exponential factor were seen at 45.0 kJ/mol and 4.769 (1/see), respectively, in step 1 for T_5_ pellets. These results agreed well with the Fang, Shi [114] research, even the different feedstock. The highest *E*_*α*_ was 632.065 kJ/mol in step 3, while the maximum *A* was 54.666 (1/s) during the combustion of the T_5_ pellet, which might be a cellulose and volatile matter decomposition. Overall, the observed results revealed that the pre-exponential factor and activation energy were correlated, which means if *A* was higher, *E*_*α*_ was also higher (Table 4). In brief, the diversity of kinetic parameters quantity attached depends on biomass pseudo-components decompositions.

The pre-exponential factor (A) fluctuation occurred due to biomass combustion reaction chemistry, such as a surface reaction or complex reactions. When *A*>109 (1/s) happened in a simpler complex reaction, but *A*<109 (1/s), the reaction did not depend on the surface [115]. According to the results for T_1_ pellets, steps 1 and 4 were independent on the surface, while steps 2 and 3 represent simpler complex reactions (Table 5). Alternatively, steps 1 and 4 signify no dependent reaction on the surface, while steps 2 and 3 illustrate the activated complex reaction for the T_5_ pellet (Table 5). Alternatively, the activated complex's limited reactions were termed when *A* between 1010 and 1012 (1/s) [97].

#### 3.3.3 Thermochemical reaction dimensionality

For the assessment of reaction dimensionality (*n*) based on model-based kinetic expressions, the reaction mechanism was considered according to 20 °C/min heating rate, while temperature ranges from 25 to 1200 °C. The consecutive reaction mechanism can observe for all steps assessing the dimensionality (*n*) that its values were higher than the unity (*n* > 1) for steps 3 and 4 (Table 5) [116]. Alternatively, steps 1 and 2 were the non-integer dimensionality (less than a unit or equal to zero) that can result from the size and shapes of reaction particles [117]. Changes in *E*_*α*_ values through numerous elemental phases were linked to dimensional variations throughout the steps. Additionally, the *n* and *E*_*α*_ are likely to vary when fundamental changes occur in one element to another stage.

Overall results (Table 5) showed that both pellets' reaction types were similar. However, the reaction order value differed. Therefore, the reaction types were not significantly influenced by the additives blends pellet (T_5_).

#### 3.3.4 Kinetic reaction model

The kinetic mechanism evaluated for various reaction models is listed in Table 2. A single kinetic model function of F2, D3, Cn, and Fn models can reproduce the overall process for the observed results (Table 5).

The F2 model best described the primary combustion steps (peak 1) of both pellets types; this reflected cellulose decomposition [118]. In this stage, the reaction type was phase interfacial second order. This result agrees with Huang, Zhang [111]. In addition, for step 2, the observed diffusion-controlled process may be involved and modelled as D3 (3-D diffusion), while the function is *f(α) = 1.5* $e^{\frac{2}{3}}$*/(1-*$e^{\frac{1}{3}}$*),* which was noted in the combustion complex reaction. These results agree with Várhegyi, Chen [119]. As a solid carbonaceous biomass, wheat straw pellets decomposed when heated, causing gaseous by-products to float to the surface of the particles. Diffusion frequently causes the reaction rate to slow down and could restrict the reaction rate.

For step 3, the C*n* kinetic model represents the most suitable model for describing the combustion process through the autocatalyst model. The suitability of the autocatalyst model was for the description of the nucleation-driven process. This reaction process significantly reduced the growth rate because of intensified fragmentation and emission of volatiles [120]. These types of reactions accelerate the reaction mechanism. The final step (peak 4) was the carbonisation region, where the leftover residue was biochar or carbon-enriched ash. This step was titled phase interfacial ^n^th order reaction; that differential function was *f(α)* = $e^{n}$ (Table 2). Overall, the reaction order models were well suited to describe the combustion reaction mechanism of lignocellulosic biomass (WSP). Therefore, it can be concluded that several steps occurred due to the decomposition of biomass components and complex reaction effects.

### 3.4 Relationship between degree of conversion and kinetic parameters

**Fig. 3:**
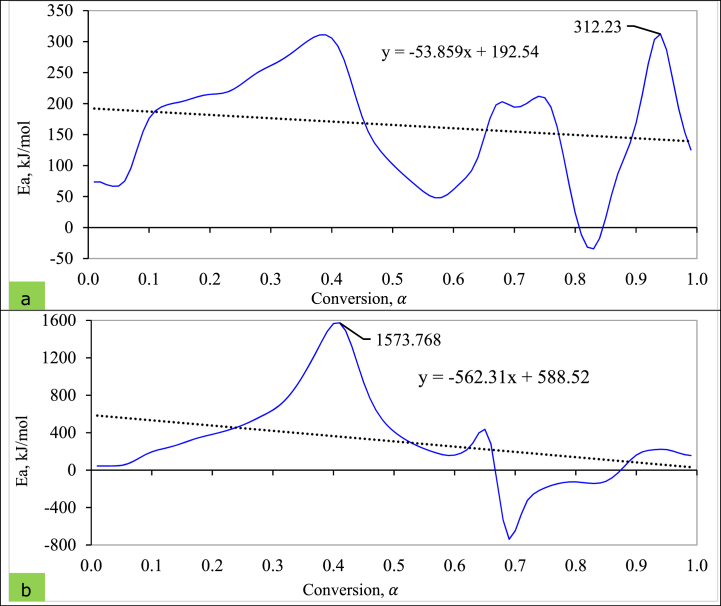
Dependence of conversion degree and activation energy for (a) T_1_ and (b) T_5_ pellet.

Biomass various compositional variations might suggest a complex combustion reaction that results in *E*_*α*_ differences. The relationship between activation energy and conversion degree is demonstrated in Fig. 3 (a, b).

The activation energy (*E*_*α*_) fluctuated in both pellet's types, regarding the reaction degree (α). This fluctuation profile exhibits various endothermic and exothermic picks, resulting in energy absorption and release during different reactions [6]. Moreover, the *E*_*α*_ increases (+value) signifies the endothermicity with conversion, while the activation energy decreased (-value) indicates exothermic reactions of the combustion process [121]. This trend was observed in the study of Thakur, Varma [104], which suggests that a complicated series of processes occurred during the combustion of WSP, and a significantly less amount of cellulose and hemicellulose remained.

The maximum activation energy for T_1_ pellets found was 312.23 kJ/mol, attributed to the energy absorbed during the degradation of hemicellulose and cellulose (Fig. 3a). At the same time, the minimum *E*_*α*_ was (−) 34.307 kJ/mol representing energy release in lignin breakdown. Alternatively, the peak *E*_*α*_ value was 1573.768 kJ/mol with a conversion degree of 0.41 for T_5_ pellets (Fig. 3b). The maximum negative value of *E*_*α*_ was (−) 738.795 kJ/mol at the degree of conversion point at 0.69. Overall, both pellets followed the same fluctuations, but the linear trendline differed (Fig. 3). Sharma, Pandey [122] noted that the connection between the *E*_*α*_ and A were correlated, which did not support the present study results and could be a variation of the analysis method.

**Fig. 4:**
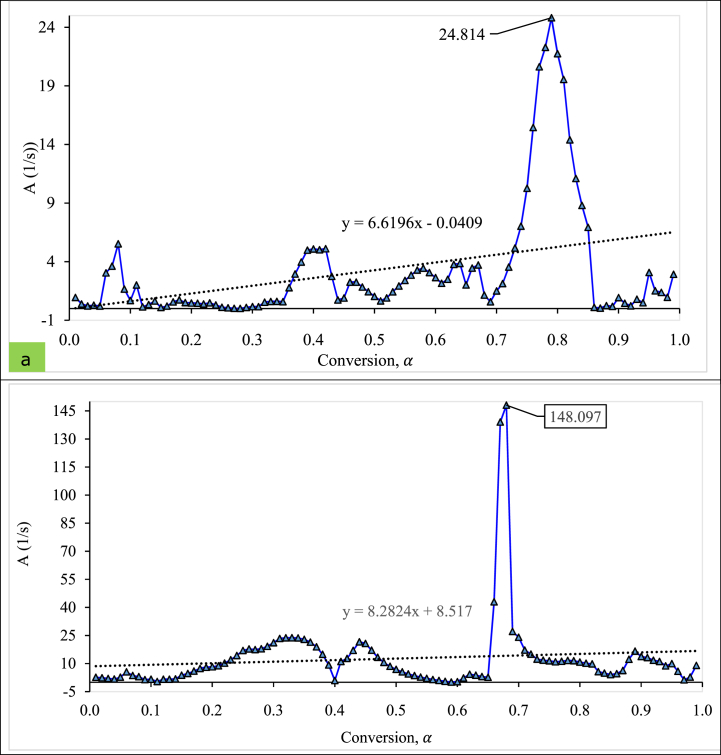
Conversion degree vs Pro-exponential factor plot for (a) T_1_ and (b) T_5_ pellet.

Fig. 4 (a, b) depicts the relationship between the pre-exponential factor (*A*) versus the conversion degree (*α)*. As the degree of conversion rose for 0.1 to 1.0, the activation energy fluctuated with the values in both pellets. The highest *A* were 24.814 and 148.18 (1/s) for T_1_ and T_5_, respectively. Inversely, (−)0.27 and (−)0.11 (1/s) were the minimum pre-exponential factors for T_1_ and T_5_ pellets. The observation indicates that exothermic (-value) and endothermic (+value) reactions happened during conversion [123]. The current model-free analysis follows the various degree of conversion ranges, which are commonly acknowledged, including lower conversions (α < 0.1), medium conversions (0.1 ≥α ≤ 0.20), maximum conversion (0.20 > α ≤ 0.95), and at higher conversions (α > 0.95). The results obtained in the current study also align with the findings reported by Mandal, Mohalik [90].

The profiles and linear trendline of two pellets (T_1_ and T_5_) followed the fluctuation, but the trend was not similar. From the figure, the coefficient of determination (R^2^) value was found to be relatively lower (∼0.15); consequently, there was no significant correlation between the conversion degree and the pre-exponential factor. The pre-exponential factor (*A*) dependency on the degree of conversion followed the same trend, which supported the study of Muravyev, Pivkina [121]; however, the varying *A* value might be the different analysis methods.

### 3.5 Analysis of thermodynamic parameters

Entropy, enthalpy, and Gibbs free energy are the thermodynamic properties that help decide a reactor's heating and cooling requirements/arrangement [124]. Table 6 displays the thermodynamic parameters, which were determined using empirical equations. To determine the entropy, enthalpy, and Gibbs free energy, the *E*_*α*_ and *A* taken from the *NETZSCH* program at 20 °C/min heating rates. According to the results (Table 6), the average activation energy and enthalpy difference was approximately 5 kJ/mol. This trait was consistent with Kaur, Gera [98]. Both pellets in peak 1 (first zone) gave the lowest value for all thermodynamics parameters; this result agreed with the research output of Naqvi, Ali [125] and Gajera, Tyagi [126]. The reaction is endothermic and releases energy, as seen by the reaction's negative enthalpy value. In the T_1_ pellets, the highest enthalpy *(*Δ *H*) and change in Gibbs free energy (Δ *G*) were 268.747 kJ/mol and 308.100 kJ/mol, respectively, for peak 2.

**Table 6:** Thermodynamic parameters for combustion of wheat straw pellet at 20 °C/min heating rate.

Pellets	Items	Peak 1	Peak 2	Peak 3	Peak 4
T_1_	Tm, °K	345.15	570.15	630.15	715.15
	$E_{\alpha }\text{,}$ kJ/mol	45.00	273.488	189.782	164.723
	*A*, (1/s)	117.801	8.01E+9	1.53E+6	2.40E+4
	Δ*H*, kJ/mol	42.130	268.747	184.542	158.776
	Δ*G*, kJ/mol	116.268	308.100	273.405	285.090
	Δ*S*, kJ/mol.K	−0.214	−0.069	−0.141	−0.176
T_5_	Tm, °K	345.15	550.15	580.15	680.15
	$E_{\alpha }\text{,}$ kJ/mol	45.03	418.94	632.07	115.47
	*A*, (1/s)	1.172E+2	1.75E+16	5.509E+23	1.103E+3
	Δ*H*, kJ/mol	42.16	414.36	627.24	109.81
	Δ*G*, kJ/mol	116.32	385.38	513.68	247.10
	Δ*S*, kJ/mol.K	−0.21	0.05	0.20	−0.20

Note: Tm = Peak temperature at a maximum reaction rate.

Peak 1 = Mass losses occurred due to the moisture.

Peak 2 = Mass losses due to oxidative degradation (i. e., volatile released and then burned)

Peak 3 = Mass losses oxidative degradation (decompose of cellulose)

Peak 4 = Mass losses combustion of the remaining char.

In addition, all negative values for entropy (Δ*S*) suggest that T_1_ pellets combustion experiences minor physical and chemical changes and trends towards thermodynamic equilibrium [127]. Alternatively, for T_5_ pellets, the entropy of the first (peak 1) and the last reaction (peak 4) was negative; however, the 2nd and 3rd zone values were positive. This finding indicates that the reaction did not reach equilibrium due to the activated complex formation's association reaction mechanism [128,129].

## 4 Limitations of the study

The research examined the thermokinetics of wheat straw pellet combustion by TGA. For more precision and accurate results need to develop a mathematical model. Also, simulation helps to optimise the influencing factors of combustion., which did not consider in this study. Future research should include model development based on machine learning, a hybrid intelligence grogram for validation and sensitivity analysis.

The WSP decomposition was best fitted with a model-based method for analysis and multi-point reaction. However, the researcher commonly used both model-free and model-based techniques. Further, several studies considered the single-stage reaction. Therefore, the present study result needs to investigate in-depth. Further, the results need to investigate and validated for another lignocellulosic biomass.

## 5 Conclusions and future perspectives

In this study, the combustion characteristics of two wheat straw pellets were analyzed using a thermogravimetric analyser. The kinetic characteristics of the combustion process of wheat straw pellets were determined using TGA in combination with a model-based method. The WSP thermokinetic reaction followed the four steps for both pellets A → B (dehydration and desorption), C → D (Oxidation), D → E (combustion) and E →F (burnout). The activation energy for the regions I, II, II, and IV were 45.0, 273.488, 189.782, and 164.723 kJ/mol, respectively, for the T_1_ pellet, alternatively 45.034, 418.935, 632.065, and 115.470 kJ/mol of E a for T_5_ pellet. According to model-based techniques, the WSP degradation mechanism was best fitted with reaction models such as Cn, Fn, F2 and D3. At the same time, the variation of the *E*_*α*_ pointed to complex combustion reactions. The *E*_*α*_ value thus indicates the auto-catalyst as the dominant process for T_5_, while diffusion control (Jarden model) was for T_1_. In addition, the *E*_*α*_ and *A* were increasing and decreasing simultaneously with the conversion rate, indicating that the WSP composition followed the exothermic and endothermic processes. The entropy of the T_1_ pellet followed the equilibrium reactions, but the T_5_ pellet observed the non-equilibrium reaction. Hence, based on the thermodynamic parameter, the additive blended pellets had no significant impact on the reaction process. The kinetic reaction model, kinetic properties and thermodynamic parameters can be used to design and fabricate combustion and gasifier reactors as well as optimise the process conditions. The research results confirmed that the WSP can be used as a bioenergy feedstock and promoted and applied as biomass pellet fuels. Further, the activation energy and pre-exponential factor are important for CFD model development.

## Author contribution statement

Bidhan Nath: Conceived and designed the experiments; Performed the experiments; Analyzed and interpreted the data; Contributed reagents, materials, analysis tools or data; Wrote the paper.

Guangnan Chen; Les Bowtell: Conceived and designed the experiments; Wrote the paper.

Elizabeth Graham: Performed the experiments; Analyzed and interpreted the data.

## Data availability statement

Data will be made available on request.

## Additional information

No additional information is available for this paper.

## Declaration of competing interest

The authors declare that they have no known competing financial interests or personal relationships that could have appeared to influence the work reported in this paper.
